# A Crucial Aftershock in Pulmonary Tuberculosis Survivors: A Case Report

**DOI:** 10.7759/cureus.20986

**Published:** 2022-01-06

**Authors:** Prashant Ahlawat, Prateek Upadhyay

**Affiliations:** 1 General Medicine, Government Medical College & Hospital, Chandigarh, Chandigarh, IND; 2 Anaesthesia and Intensive Care, Government Medical College & Hospital, Chandigarh, Chandigarh, IND

**Keywords:** tb sequalae, hemoptysis, necrotizing pneumonia, rasmussen’s aneurysm, tuberculosis

## Abstract

Pulmonary tuberculosis is widely prevalent, and the survivors of this disease often present to healthcare facilities with long-term sequelae of the disease. Presented here is a case of a 25-year-old male who presented with concerns of fever, cough with expectoration, and blood in sputum. The patient was managed as per protocol in suspicion of necrotizing pneumonia and re-activation of tuberculosis (TB) as suggested by investigations. The hemoptysis gradually increased over time. With suspicion of a vascular aneurysm and in view of increasing hemoptysis, an early high-resolution computed tomography (HRCT) scan of the chest and a computed tomography (CT) scan of the bronchial angiography were performed. A diagnosis of Rasmussen’s aneurysm was made radiologically, and this rare and under-reported sequelae of TB in contemporary times was brought into notice. A holistic and multi-disciplinary approach involving emergency medicine physicians, internists, anesthesiologists, critical care physicians, pulmonologists, and radiologists can ensure optimal outcomes for such cases in hospital setups if timely intervened.

## Introduction

Pulmonary tuberculosis (PTB) is widely prevalent in developing countries like India. Even after anti-tubercular treatment, it is not uncommon for survivors of tuberculosis (TB) to present to healthcare facilities with long-term sequelae of the disease. A thorough relevant workup should be performed to reveal any underlying pathology that could require immediate intervention while ensuring emergency resuscitation and stabilization. A multi-disciplinary approach needs to be instituted for optimal management in such cases. We present here an under-reported case of Rasmussen’s aneurysm, highlighting the importance of timely management and discussing the treatment plan.

## Case presentation

A 25-year-old male presented to emergency medicine with concerns about fever, cough with expectoration, and blood in the sputum. Fever was documented by the patient to be around 38°C for 10 days. It occurred more in the evening and was relieved by medicine (acetaminophen). He complained of productive cough for 10 days. The expectorant was greenish, three to four teaspoons full and throughout the day. The patient reported a history of pulmonary tuberculosis (PTB) two years ago with no dissemination, for which he was treated for six months with all-oral anti-tubercular treatment (isoniazid, rifampin, ethambutol, and pyrazinamide). His sputum smear was tested negative, and he was labeled as having recovered from TB a year and a half ago. His appetite and weight had also increased, and his symptoms had resolved. He denied any substance abuse, including intravenous drug abuse. He gave a history of weight loss, apparent from the loosening of clothes.

**Figure 1:**
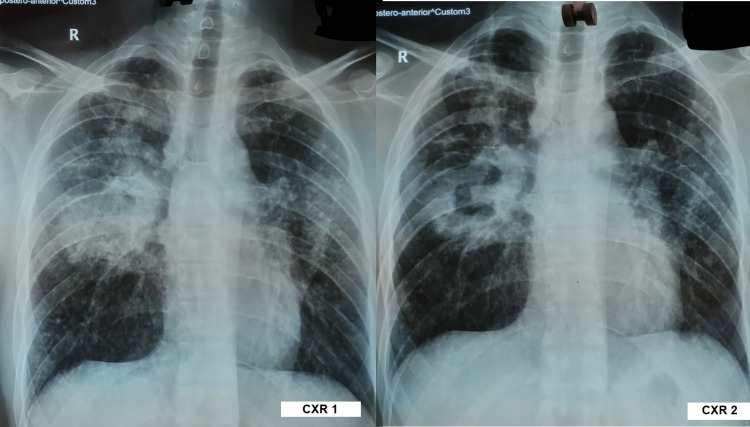
Chest x-ray images of the patient. CXR 1: chest x-ray showing ill-defined radio-opacity in right middle zone on presentation. CXR 2: chest x-ray showing an ill-defined patch of radio-opacity with area of central lucency with few patent air bronchograms after six days.

Chest x-rays (CXRs) showed right middle zone radio-opacity and were suggestive of consolidation (Figure 1; CXR 1). Routine blood investigation revealed mild microcytic hypochromic anemia with predominantly neutrophilic leucocytosis. The coagulation profile was normal. His serology was negative for the human immunodeficiency virus. Sputum culture and antibiotic sensitivity were examined, and growth of *Pseudomonas aeruginosa* was found. Thus, antibiotics were upgraded accordingly, from injection ceftriaxone to injection piperacillin-tazobactam. The patient’s general condition improved in the following four days, but the hemoptysis persisted. Acid fast bacilli (AFB) were not present in the sputum, and the cartridge-based nucleic acid amplification test (CBNAAT) didn’t find any evidence of PTB. A repeat x-ray (Figure 1; CXR 2) showed a patch of consolidation along with areas of central lucency and a few patent bronchograms that indicated necrotizing pneumonia.

**Figure 2:**
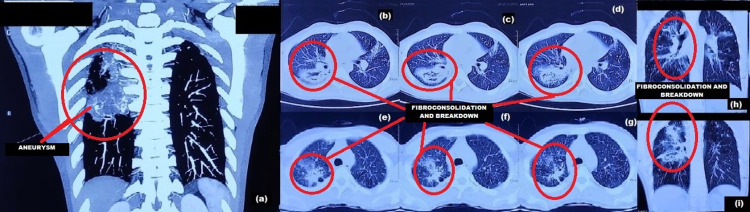
CT imaging films of the chest. (a) CT bronchial angiography showing saccular outpouching measuring 5 × 4 mm in the immediate vicinity of the cavitary lesion in the superior segment of the right lower lobe, suggestive of an aneurysm. Multiple tortuous broncho-pulmonary collaterals are seen at the right hila, the posterior segment of the right upper lobe, and the superior surface of the right lower lobe. (b) to (g) Horizontal sequential sections of high-resolution chest CT scan showing patches of fibro-consolidation having retracted and spiculated margins and with internal areas of breakdown in the apical and posterior segments of the RUL associated with fibrotic bands and pleural tags with pleural retractions. (h) and (i) Coronal sections of high-resolution chest CT showing the same finding seen in horizontal sequential sections, i.e., patches of fibro-consolidation having retracted and spiculated margins and with internal areas of breakdown in the apical and the posterior segment of the RUL associated with fibrotic bands and pleural tags with pleural retractions. CT: computed tomography, RUL: right upper lobe.

The patient improved clinically with the ongoing antibiotics, but the hemoptysis increased after two days. In view of massive hemoptysis, high-resolution computed tomography (HRCT) chest and computed tomography (CT) bronchial angiography were planned. The imaging reported an irregular patch of fibro-consolidation with internal areas of breakdown in the superior segment of the right lower lobe with a similar patch in the apical and posterior segments of the right upper lobe (RUL) and multiple centrilobular nodules. A saccular outpouching, more likely arising from the pulmonary artery (PA) and of 5 × 4 mm dimension, was seen in close approximation with the cavitary lesion in the superior segment of the right lower lobe, accompanied by hypertrophied bronchial arteries (BA) and multiple bronchopulmonary collaterals (Figure 2). The patient was managed with blood transfusion, antifibrinolytics and was referred to a higher center for urgent endovascular management.

The patient was followed up telephonically. His father informed us that he was planned for "another scan" (probably, digital subtraction angiography) and an "accompanying surgical procedure" (probably coiling in the higher center). The patient was a student, and his father was a daily-wage laborer with no other earning members in the family. Therefore, due to economic constraints (a very poor family) and risks associated with the procedure, the patient and his family did not consent to further imaging and treatment. After that, the patient was lost to follow-up.

## Discussion

India has a huge burden of tuberculosis (TB). In 2020, an estimated 10 million people contracted tuberculosis (TB) worldwide. Thirty countries with a high healthcare burden of TB accounted for 86% of the newer TB cases. Furthermore, eight countries constituted two-thirds of the total healthcare load, with India leading the count [1].

Tuberculosis (TB) is caused by the bacteria *Mycobacterium tuberculosis,* and the lungs are the most common organs to be infected [2]. The principal mode of spread of TB is via aerosolized droplets. Patients present to the healthcare facility with a constellation of symptoms, including chronic cough, weight loss, blood in sputum, an evening rise in temperature, etc. In such cases, an enquiry should be made from the patient about any contact with a TB patient. A chest x-ray should be done along with a sputum examination for AFB and nucleic acid-based tests to detect drug resistance.

In such patients presenting with mild hemoptysis, consideration needs to be put on reactivation of tuberculosis, pneumonia, and aspergillus infection of the tubercular cavity. It could also be occasionally due to chronic bronchitis or bronchiectasis in these cases. With chronic inflammation, bronchial arteries get hypertrophied, and a broncho-pulmonary arterial-venous communication can develop, which could further cause hemoptysis [3]. Mild hemoptysis in TB is usually self-limited, and it resolves while the patient is on anti-tubercular therapy (ATT). However, prolonged or massive hemoptysis should be timely diagnosed and dealt with. Since life-threatening hemoptysis is usually arterial, it requires immediate intervention [4]. Massive hemoptysis is fatal in 50% of cases [5]. In a patient with TB presenting with massive hemoptysis, bronchial arteries (BA) are the most common source for vascular complications, of which, pulmonary arteries (PA) account for <10% of hemoptysis [6,7].

In the management of these patients, the first step is to secure the airway as the patient can choke on their blood. Tracheal intubation should be performed with an endotracheal tube of the largest possible internal diameter to facilitate suctioning of blood clots. The patient should be positioned in a manner so that the bleeding lung is in a dependent position. This is to ensure that the other lung is ventilated adequately to deliver maximum tissue oxygenation. A comprehensive clinical examination should be supplemented by previous radiological investigations, if any, to determine the feasibility of positioning. All relevant investigations should be ordered promptly, and adequate blood products need to be arranged and transfused as indicated.

This case was diagnosed as case of Rasmussen's aneurysm. It is an “inflammatory pseudo-aneurysmal dilatation of a branch of a pulmonary artery (PA) adjacent to a tubercular cavity [8].” It is a rare phenomenon resulting from a weakened wall of the pulmonary artery with a prevalence of merely 5% [9]. It must not escape attention and consideration since it has a propensity to rupture and lead to massive hemoptysis and death [7]. In a pioneer case series that conducted autopsies of 1,114 pulmonary tuberculosis patients with cavitary lesions, it was found that 45 cases had an associated pulmonary arterial aneurysm, comprising 4% of the total cases studied. Thirty-eight of these 45 cases, i.e., 84.4%, were responsible for fatal hemoptysis [10].

In a study that was performed to assess the reduction in the severity of bleeding in cases with sub-massive hemoptysis, it was observed that tranexamic acid (TXA) can be used as a bridging therapy until a definitive intervention is available as it decreases the severity of hemoptysis [11]. In another randomized controlled trial that enrolled patients with mild to sub-massive hemoptysis of various etiologies, a comparison between treatment with nebulized TXA and placebo (normal saline) was performed. A significant decrease in blood expectoration was observed starting from day two, and the resolution was observed by day five, with adverse effects [12].

In the described case, the patient was managed with antibiotics, anti-fibrinolytic (tranexamic acid), and blood transfusion. A CT bronchial angiography was performed promptly, which was suggestive of saccular outpouching of the artery, suggestive of an aneurysm near the cavitary lesion. The origin of the aneurysm appeared to be from the pulmonary artery. The patient was stabilized and was referred to a higher center for urgent radiological intervention. Further treatment options include embolization of the pulmonary or systemic vessels. If it is not controlled with this, the patient may require a lobectomy. If implemented timely, endovascular management has been shown to have a 100% success rate in managing pulmonary artery aneurysms and pseudoaneurysms [13]. This case brings into light a rare phenomenon that is often unheeded and disregarded [9]. Firstly, the diagnosis of Rasmussen's aneurysm in a young adult with no other co-morbidities, who was labeled to have recovered completely from pulmonary tuberculosis after compliantly adhering to the course of anti-tubercular treatment as per a negative sputum smear and then presenting with such a deathly complication highlights the importance of paying heed to the unheeded complications, particularly in this age group. Secondly, in cases with intractable, persistent and increasing hemoptysis, a bronchial vessel pathology is suspected instead of a pulmonary vessel since the bronchial artery has a higher pressure. In resource-limited countries like India, and for patients with financial constraints, it is not possible to go for a repeated CT scan, especially with contrast. The very skilled radiologist in our case discussed the films with a senior consultant and was able to trace the pulmonary artery despite the plethora of collaterals in the area. Thirdly, the economical burden of TB, which compelled the patient to abandon the treatment raises a very pertinent question if we need to re-evaluate the policy-making on an administrative level and if we are able to do justice to the under-privileged patients, particularly in a resource-limited setting.

## Conclusions

Patients with old, treated pulmonary tuberculosis often present to healthcare with sequelae of the disease. In cases with hemoptysis, a failure of timely diagnosis and management can be fatal. Due attention needs to be given to the possibility of Rasmussen's aneurysm in view of its high case fatality. A holistic and multi-disciplinary approach involving emergency medicine physician, internist, anesthesiologist/critical care physician, pulmonologist and radiologist can ensure optimal outcomes in hospital setups.
